# Elections

**Published:** 1888-12

**Authors:** 


### ELECTIONS.

### AMERICAN ASSOCIATION.

Saratoga was chosen as the next place of meeting. The following officers were chosen:

*President*—Chas. R. Butler, Cleveland, Ohio.

*First Vice-President*—A. W. Harlan, Chicago, Ill.

*Second Vice-President*—S. A. White, Savannah, Ga.

*Corresponding Secretary*—F. A. Levy, Orange, N. J.

*Recording Secretary*—Geo. H. Cushing, Chicago, Ill.

*Treasurer*—N. H. Fuller, St. Louis, Mo.

*Executive Committee* (New Members)—E. T. Darby, Philadelphia; Geo. W. McElhany, Columbus, Ga.; I. N. Crown, Chicago; Frank Abbott, New York.

### SOUTHERN ASSOCIATION.

Galveston, Texas, was chosen as the next place of meeting.

#### OFFICERS.

*President*—J. Y. Crawford, Nashville, Tenn.

*First Vice-President*—John C. Storey, Dallas, Texas.

*Second Vice-President*—Wm. N. Morrison, St. Louis, Mo.

*Third Vice-President*—J. S. Thompson, Atlanta, Ga.

*Corresponding Secretary*—D. R. Stubblefield, Nashville, Tenn.

*Recording Secretary*—M. C. Marshall, Little Rock, Ark.

*Treasurer*—H. A. Lawrance, Athens, Ga.

*Executive Committee* (one year)—Dr. Dyer, Dr. W. R. Clifton, Waco, Texas; (two years), Dr. G. S. Staples, Sherman, Texas; Dr. B. H. Catching, Atlanta, Ga.; (three years), Dr. H. E. Beach, Clarksville, Tenn.; Dr. H. I. McKellops, St. Louis, Mo.

